# CC Chemokine Receptor 4 (CCR4) as a Possible New Target for Therapy

**DOI:** 10.3390/ijms232415638

**Published:** 2022-12-09

**Authors:** Joanna Bogacka, Katarzyna Pawlik, Katarzyna Ciapała, Agata Ciechanowska, Joanna Mika

**Affiliations:** Department of Pain Pharmacology, Maj Institute of Pharmacology, Polish Academy of Sciences, 12 Smetna Street, 31-343 Krakow, Poland

**Keywords:** CCR4, CCL17, CCL22, CCL2, chemokines, opioids, neuropathy

## Abstract

Chemokines and their receptors participate in many biological processes, including the modulation of neuroimmune interactions. Approximately fifty chemokines are distinguished in humans, which are classified into four subfamilies based on the N-terminal conserved cysteine motifs: CXC, CC, C, and CX3C. Chemokines activate specific receptors localized on the surface of various immune and nervous cells. Approximately twenty chemokine receptors have been identified, and each of these receptors is a seven-transmembrane G-protein coupled receptor. Recent studies provide new evidence that CC chemokine receptor 4 (CCR4) is important in the pathogenesis of many diseases, such as diabetes, multiple sclerosis, asthma, dermatitis, and cancer. This review briefly characterizes CCR4 and its ligands (CCL17, CCL22, and CCL2), and their contributions to immunological and neoplastic diseases. The review notes a significant role of CCR4 in nociceptive transmission, especially in painful neuropathy, which accompanies many diseases. The pharmacological blockade of CCR4 seems beneficial because of its pain-relieving effects and its influence on opioid efficacy. The possibilities of using the CCL2/CCL17/CCL22/CCR4 axis as a target in new therapies for many diseases are also discussed.

## 1. Chemokines and Their Receptors

**Figure 1:**
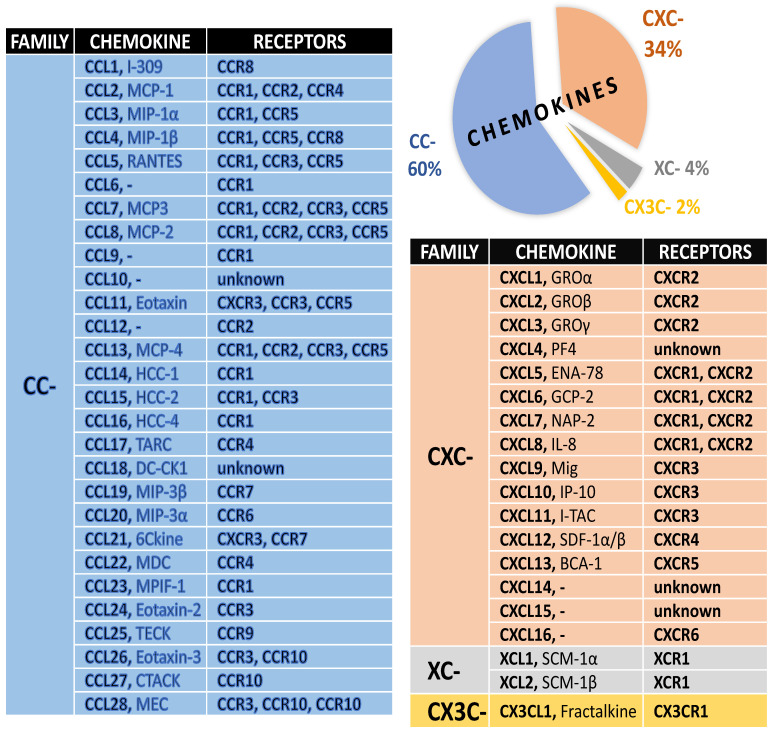
Classification of chemokine family—chemokines (systematic and original names) and their receptors. Abbreviations of original names of chemokines: BCA-1, B-cell-attracting chemokine 1; CTACK, cutaneous T-cell-attracting chemokine; DC-CK1, dendritic-cell-derived CC chemokine 1; ENA-78, epithelial-cell-derived neutrophil attractant 78; GCP, granulocyte chemotactic protein; GRO, growth-related oncogene; HCC, haemofiltrate CC chemokine; IL, interleukin; IP-10, interferon-inducible protein 10; I-TAC, interferon-inducible T-cell alpha chemoattractant; LEC, liver-expressed chemokine; LCC-1, liver-specific CC chemokine-1; MCP, monocyte chemoattractant protein; MDC, macrophage-derived chemokine; MEC, mammary-enriched chemokine; Mig, monokine induced by interferon γ; MIP, macrophage inflammatory protein; MPIF, myeloid progenitor inhibitory factor; NAP, neutrophil-activating peptide; PF4, platelet factor 4; RANTES, ‘regulated on activation, normally T-cell-expressed and -secreted’; SCM-1α/β, single C motif-1 α/β; SDF, stromal-cell-derived factor; TARC, thymus- and activation-regulated chemokine; TECK, thymus-expressed chemokine.

The 20 receptors for chemokines have been described and divided into four main families: CC, CXC, XC, and CX3C [1]. Each of these receptors are bound to a G protein at the C-terminus and interacts with its intracellular elements. The structure of these receptors includes a single helical polypeptide chain that crosses the cell membrane seven times with two disulfide bridges located between the N-terminal domain and the second extracellular loop and between the first and third extracellular loops [2,3]. Approximately 50 ligands of chemokine receptors have been identified and classified into four basic families: CC, CXC, XC, and CX3C [1]. Some chemokines have pleiotropic properties and activate different receptors (Figure 1), including CCL3/CCR1, CCR5; CCL4/CCR5, CCR8; CCL5/CCR1, CCR3, CCR5; CCL7/CCR1, CCR2; CCR3; CCR5; and CCL11/CCR2/CCR3/CCR5, and some chemokines activate only one receptor, such as CX3CL1-CX3CR1, CXCL25-CXCR9, and CXCL13-CXCR5 [3]. After binding with their ligand, chemokine receptors activate a cascade of intracellular signaling pathways, e.g., mitogen-activated protein kinase (MAPK), phospholipase C (PLC), and phosphatidylinositol 3-kinase (PI3-K), which lead to a wide range of cellular processes, such as chemotaxis, adhesion, cell activation or cell polarization, which amplify the production of cytokines [4,5,6].

Chemokine receptors play key roles in several diseases, including allergies, atherosclerosis, viruses, cancer, various infections, and inflammation [7]. Research indicates that immune cells and glia (microglia and astrocytes) express most chemokine receptors in the central nervous system, but many of these receptors are also located on neurons [8,9,10,11,12].

**Figure 2:**
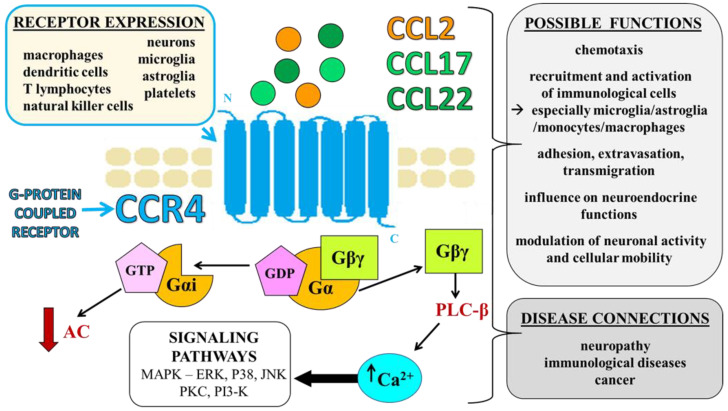
CCR4—mechanisms of action, possible roles, cellular expression, and disease connections. Abbreviations: chemokine receptor 4—CCR4; chemokine (C-C motif) ligand 22 (CCL2); chemokine (C-C motif) ligand 17 (CCL17); chemokine (C-C motif) ligand 22 (CCL22).

The involvement of several chemokine receptors, such as CCR1 [13,14], CCR2 [4,15,16], CCR5 [17,18], CCR8 [19], CXCR2 [9], CXCR3 [20], CXCR4 [21,22], and XCR1 [23], has been documented in nociception and neuropathic pain of different origins (Table 1). Blockade of these receptors relieves pain and shows beneficial effects in numerous diseases, such as colitis [24], rheumatoid arthritis [25,26], allergies [27], HIV [28,29], leukemia [30], and multiple sclerosis [31]. Because of the important role of chemokines and chemokine receptors in various physiological functions and their association with many pathological conditions, these factors have become targets in the search for new treatments for several human diseases. However, the role of many of these factors is not fully known, and one of the most interesting factors of this group is the C-C chemokine receptor type 4 (CCR4). CCR4 influences the development of the inflammatory process [32], and subsequent researchers demonstrated the important role of this receptor in the processes of nociception [33,34,35], immunological diseases [36,37,38,39], and cancer [38,39,40]. Data indicate that CCL17 and CCL22 are selective CCR4 ligands [37,41]. However, it was suggested that CCL2, which was widely regarded as the major ligand of CCR2, also acted via CCR4 [42], which is interesting because these chemokines are important agents in physiology and pathology (Figure 2). Therefore, the role of this receptor and its endogenous ligands is discussed in the following chapters.

## 2. CCR4 and Its Ligands—Identification, Expression, and Functions

According to the current classification, CCR4 belongs to the CC group and was cloned from a human basophilic cell line [43,44,45]. Its presence has been demonstrated on T lymphocytes (Th2, Th17, and Tregs), platelets, natural killer (NK) cells, monocytes, macrophages, dendritic cells [37,41,46], neurons [47], microglia [48], and astroglia [41,48]. Notably, CCR4 has been found at different levels of the nervous system, such as in the dorsal root ganglia [49], spinal cord [50], and brain [51], which suggests its pivotal roles in physiology and pathology. CCR4 belongs to the family of transmembrane metabotropic receptors that respond to cell signals via G proteins [43]. CCR4 consists of a single polypeptide chain that crosses the membrane seven times. The amino terminus is extracellular, and the carboxyl terminus is intracellular. Notably, CCR4 may occur in at least two different conformational types. The major population is activated by CCL17 and CCL22, and the second population is responsive only to CCL22 [52]. The contribution of CCR4 to the pathogenesis of autoimmune encephalitis [41,50,53], multiple sclerosis [41], asthma [54], and atopic dermatitis [36] has been shown. Clinical trials revealed the important role of CCR4 in cancers, such as leukemia/lymphoma [38], breast cancer [39], and renal cancer [40]. Notably, Kiguchi et al. (2017) showed a CCR4 increase in the spinal cords of monkeys with diabetes [55], but its role in nociception was not described until 2020.

### 2.1. Selective Ligands of CCR4—CCL17 and CCL22

The gene encoding CCL17, thymus, and activation-regulated chemokine (TARC) was discovered in 1996 in the thymus on human chromosome 16q13 [37,56]. CCL17 is released by lymphocytes, peripheral blood mononuclear cells, dendritic cells, and neurons [41,57,58]. CCL17 is involved in various infectious and autoimmune diseases [57,59,60,61], inflammatory bowel disease, and atherosclerosis. This chemokine is responsible for chemotaxis and Ca^2+^ influx into cells expressing CCR4 [62].

The second selective CCR4 ligand, macrophage-derived chemokine-MDC (CCL22), is also found on human chromosome 16q13 [37,56] and shows a 37% similarity at the amino acid level with CCL17 and a high affinity for CCR4 [56,62,63]. Monocytes release CCL22, which acts as a chemoattractant for T cells, NK cells, and dendritic cells [41,64]. Similar to CCL17, this chemokine is also highly expressed in the thymus and bone marrow cells. Increased CCL22 expression has been observed in allergies and inflammatory skin responses [64] in the lungs of patients with allergic asthma and atopic dermatitis [36,54]. The overexpression of CCL22 prevented autoimmune β cell destruction and the development of type 1 diabetes in a mouse model [65]. Activated M2 phenotype macrophages associated with the Th2 response and tissue regeneration produce CCL22 in large amounts [66,67]. CCL22 controls Treg recruitment in various human tumors [68,69].

Notably, CCL17 and CCL22 show different properties in receptor desensitization, and CCL22 predominates over CCL17 in the ligand-induced internalization of CCR4 [52,70]. Increased levels of both chemokines were demonstrated in the serum of fibromyalgia patients [71]. Recent pharmacological studies showed that a single intrathecal injection of CCL17 and CCL22 induced pain-related behavior in naive mice [33].

### 2.2. Nonselective Ligand of CCR4—CCL2

CCL2 also activates CCR4 [42,72,73]. Due to the pleiotropic properties of this chemokine, it is one of the best studied. CCL2, also known as monocyte chemoattractant protein-1 (MCP-1), is a chemotactic and activating factor for monocyte differentiation into macrophages after penetration into tissue, memory T lymphocytes, and NK cells. The main source of CCL2 is monocytes/macrophages [74], but it is also expressed by endothelial and epithelial cells, fibroblasts, smooth muscle, astroglia, and microglia [75,76]. This chemokine is associated with the increased growth and progression of breast, ovarian, and prostate cancer [77,78,79]. CCL2 may be a therapeutic target for the treatment of various diseases, including multiple sclerosis [10], rheumatoid arthritis [80], atherosclerosis [81], and diabetes [82]. An increased level of CCL2 in various structures of the nervous system was observed in an animal model of neuropathic pain, which was associated with the activation of glial cells [17,83,84]. Pharmacological studies showed that intrathecal injections of CCL2 induced pain-related behaviors in naïve mice, long-lasting thermal hypersensitivity [85], and microglial activation. Notably, intrathecal injections of a CCL2-neutralizing antibody effectively reversed pain-related behavior in neuropathic mice [86,87]. Although the well-known pronociceptive properties of this chemokine are primarily associated with CCR2 [4,15,16], recent research provides evidence that CCL2/CCR4 signaling is also involved in these effects [35].

## 3. Pharmacological Blockade of CCR4 and Its Ligands Using CCR4 Antagonists and Blocking Antibodies

Purandare and Somerville (2006) described four main groups of CCR4 antagonists distinguished on the basis of their chemical properties: aryl sulfonamides, substituted aminoheterocycles, thiazolidinones, and lactams [88,89]. There have been many reports of CCR4 antagonist development, primarily from two groups. The first group is a collection of lipophilic heteroarenes from Bristol Myers Squibb, Astellas, and Daiichi Sankyo, and the second group is aryl sulfonamides produced by various companies, such as Astra Zeneca, Ono, and GlaxoSmithKline [90,91,92,93,94,95,96]. However, most of these antagonists are not clinically approved due to their complex mechanism of action. The indazole aryl sulfonamide GSK 2239633 has been used in phase one clinical trials in asthma and allergic inflammation models, but these studies were terminated due to multiple problems with their mechanisms of action [41,93].

C021 is often used as a potent CCR4 antagonist in experimental studies. Its characteristics indicate that it is a cell-permeable diaminoquinazoline compound ([1,4’-bipiperidine]-1’-*N*-cycloheptyl-6,7-dimethoxy-4-quinazolinamine dihydrochloride). C021 inhibited CCL22/CCR4-mediated chemotaxis in murine and human cultures [96,97]. C021 reduced microglial activation and proinflammatory cytokine upregulation in animal models of hepatic encephalopathy [42] and neuropathic pain [33,35]. Moreover, it was recently discovered that two new substances, namely AZD-2098 and AZD-1678, are CCR4 antagonists, and research aiming to clarify their characteristics is still ongoing [98].

**Figure 3:**
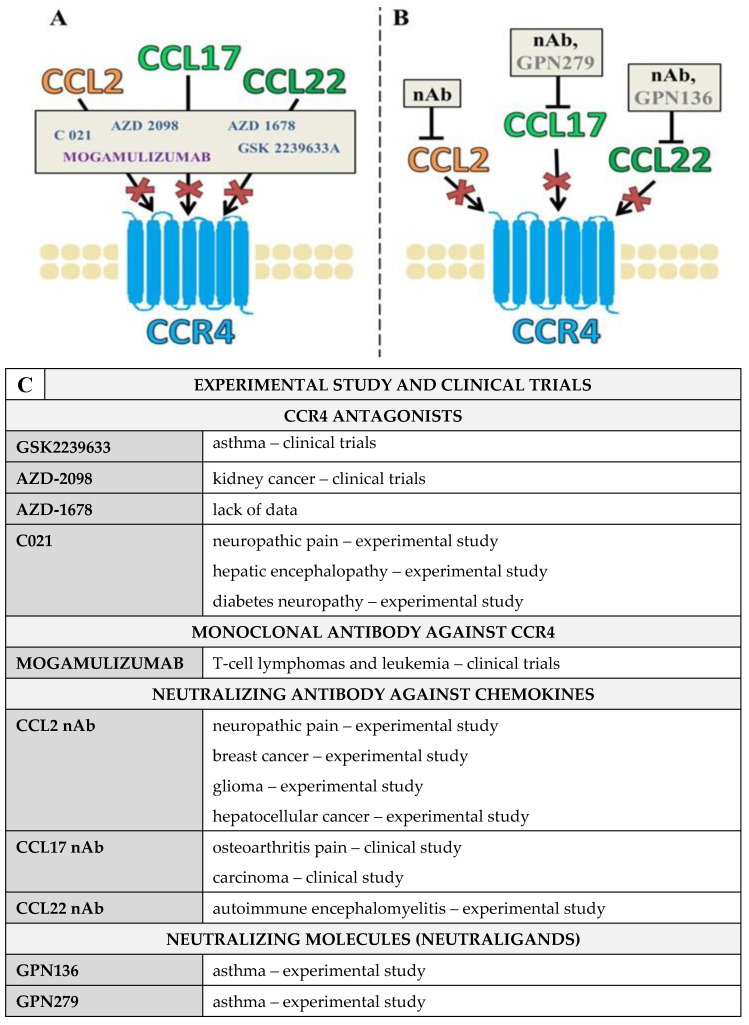
CCR4 as a possible new target for therapy-available pharmacological tools for study. (**A**) blockade of the CCR4 by its antagonists (AZD-2098 [98], AZD-1678 [98]; C021 [33,34,35,42]; GSK 2239633 [41,93]) and by monoclonal antibodies against CCR4 (mogamulizumab [37,38,100,101,102]); (**B**) neutralization of CCR4 ligands by antibodies (CCL2 [104,107,108,109], CCL17 [105,110,111], CCL22 [106]) and using neutralizing molecules of its ligands (GPN279—target CCL17 and GPN136 -target CCL22 [103]). (**C**) the table summing the experimental study and clinical trials. Abbreviations: chemokine receptor 4—CCR4; chemokine (C-C motif) ligand 22 (CCL2); chemokine (C-C motif) ligand 17 (CCL17); chemokine (C-C motif) ligand 22 (CCL22); neutralization antibodies (nAb).

Research into the development of monoclonal antibodies against CCR4 is also in progress [99,100]. Clinical trials of the humanized CCR4 antibody mogamulizumab were approved in Japan for the treatment of T-cell lymphomas, leukemia [100,101], and advanced solid tumors [100]. The mechanism of action of mogamulizumab involves CCR4-positive Treg cells. However, inhibition of Treg cell function may produce side effects, including skin rash due to a reduction in the number of these cells, which emphasizes their multidirectional mechanism of action [102]. Mogamulizumab may also have applications in the treatment of allergic diseases, such as asthma and atopic dermatitis [37]. However, this medication remains at an early stage of clinical use and requires much more extensive research focused on application and in combination with other drugs to maximize its benefits and minimize its negative effects [41] (Figure 3).

### CCL2/CCL17/CCL22 Neutraligands and Neutralizing Antibodies

Chemokine-neutralizing molecules (neutraligands) that target CCL17 and CCL22 have been created [103]. In vitro experiments showed that neutraligands, such as GPN279 and GPN136, inhibited CCL17- and CCL22-induced intracellular calcium responses, endocytosis, and T-cell migration [103]. Neutraligands inhibited inflammation in a mouse model of asthma [103]. The injection of a neutralizing antibody (nAb) to CCL2 into animals with developed neuropathic pain reduced hypersensitivity. CCL2 neutralization was effective against hepatocellular cancer in a mouse model [104]. The blockade of CCL17 using an antibody decreased the proliferation of carcinoma HeLa and SiHa cells [105], and anti-CCL22 treatment ameliorated the development of experimental autoimmune encephalomyelitis [106]. Due to the beneficial properties of the neutraligands and neutralizing antibodies against CCL2, CCL17, and CCL22, these agents seem especially important targets for future therapies (Figure 3).

## 4. CCR4 and Nociceptive Processes

Despite the significant progress in this field in recent decades, the effectiveness of treatments for neuropathic pain and the available pain medications are not satisfactory. The complex pathomechanisms of neuropathic pain directly affect the efficacy of pharmacotherapy. The International Association for the Study of Pain defines neuropathic pain as pain that arises from a “lesion or disease of the somatosensory nervous system”, and it is divided into central or peripheral pain depending on the location of the impairment. While available methods control inflammatory pain, the possibility of relieving neuropathic pain remains an issue and requires the development of new effective analgesics. Understanding the mechanisms of this pain, with a particular emphasis on the contributions of immunological factors, creates an opportunity to identify new methods for more accurate pharmacotherapies for neuropathy.

Interactions between the immune and nervous systems are crucial in neuropathic pain development and persistence. A disturbance in the communication between these systems may underlie many pathological conditions [112]. Growing evidence indicates that the activation of non-neuronal cells, especially glial cells, also plays a crucial role in the development and maintenance of neuropathic pain [113,114,115].

An increasing number of studies indicate that chemokines strongly modulate the pain response (e.g., CCL2, CCL5, CCL4, CCL3, and CXCL1) [14,23,109,116]. Intrathecal injections of CCR4 ligands (CCL17 and CCL22) [33,35] cause a rapid and strong hypersensitivity to mechanical and thermal stimuli as measured by von Frey and cold plate tests in naïve mice, which supports their pronociceptive properties. We confirmed the strong pronociceptive character of CCL2 [35], which is consistent with previously published results [15]. Our team demonstrated for the first time that the pronociceptive effects of all three chemokines were attenuated by a previous administration of the CCR4 antagonist C021. These results were not surprising for CCL17 and CCL22. However, we also showed that CCL2 may be a pronociceptive ligand of CCR4 since CCL2-evoked pain-related behaviors were diminished when preceded by the administration of C021. Our data found that each of these chemokines played an important role in nociceptive processes, which suggests that CCR4 blockade has therapeutic utility.

Previous studies demonstrated that both single and repeated intrathecal and intraperitoneal administration of the CCR4 antagonist C021 reduced hypersensitivity to mechanical and thermal stimuli in the von Frey and cold plate tests in rats [33] and mice [35] subjected to a chronic constriction injury (CCI) of the sciatic nerve or diabetic neuropathic pain models. The use of C021 in streptozotocin-induced diabetic neuropathy also improved locomotor activity in the RotaRod test [34]. This result is important from a clinical point of view because patients with diabetes mellitus often experience a decline in locomotor performance with the development of neuropathic pain [117,118].

Our biochemical studies were performed in rats exposed to CCI and revealed increased levels of *CCL17* and *CCL22* in the dorsal root ganglia during the development of neuropathy [33]. These results suggest that these chemokines are involved in the initiation of neuropathic pain after a peripheral nervous system injury. However, the levels of *CCL17* and *CCL22* in the spinal cords of rats and mice in this model were not changed in the early and late stages of neuropathy [33,35]. Seven days after streptozotocin administration, which induced diabetic neuropathy, the spinal levels of *CCL17* and *CCL22* were not changed [34]. However, an increase in *CCL2* in the spinal cord was observed in a neuropathic pain model, and these changes were maintained up to day 28 after injury [13]. An increase in CCL2 was also observed on day seven in a diabetic neuropathy model [34]. These results highlight the important role of the CCL2/CCR4 axis in nociception at the spinal cord level.

CCR4 may play a key role in nociceptive transmission in the peripheral nervous system, which may be related to elevated levels of *CCL17/22* [33] and *CCL2* [15] in the dorsal root ganglia. Notably, a CCR4 antagonist is more effective when administered intraperitoneally, which is not similar to other chemokine antagonists, such as RS5043930 for CCR2 [119] or maraviroc for CCR5 [18]. The results of our research suggest that CCR4 is a very interesting and unique target in the search for future painkillers.

Recent studies, including ours, suggest that an imbalance between pronociceptive factors (e.g., IL-1beta, IL-18, IL-6, and NOS2) and antinociceptive factors (e.g., IL-1RA, IL-18BP, and IL-10) released from various immune and nonimmune cells is involved in the development of neuropathic pain [14,18,120,121]. Therefore, their modulation may have therapeutic benefits. Repeated intrathecal administration of C021 reduced hypersensitivity to mechanical and thermal stimuli in CCI-exposed rats and the level of a macrophage/microglia activation marker, IBA-1, in the spinal cord and dorsal root ganglia [33] with no effect on the levels of astroglial markers (GFAP) or T lymphocytes (CD4, CD8). Intrathecal administration of C021 also reduced the level of pronociceptive interleukins, such as IL-1beta and IL-18, in the spinal cord but not in the dorsal root ganglia. Repeated intraperitoneal treatment with C021 produced similar results for spinal macrophages/microglia. C021 significantly reduced the levels of IBA-1, but not GFAP, 12 days after ligation of the sciatic nerve [35]. Notably, a lower level of CCL2 was also measured, which suggests that this chemokine plays a key role in the initiation and maintenance of neuropathy. Therefore, targeting CCR4 is a promising strategy to provide a new basis for understanding neuropathic pain pathomechanisms with potentially new therapeutic utility.

The coadministration of different drugs is often necessary for neuropathic pain therapy. Opioid analgesics are less effective in neuropathy than other types of pain [122,123,124,125], and the mechanisms of this phenomenon are poorly understood. The reduced efficacy of opioids in neuropathy, and their numerous side effects associated with the use of high doses, limit their usefulness, which is a serious clinical problem [126,127]. Many studies, including ours, demonstrated that the analgesic properties of morphine were largely influenced by activated microglial cells and the release of pronociceptive factors, such as interleukins (e.g., IL-1beta and IL-18) [123,128,129,130] and chemokines (e.g., CCL2 and CCL5) [109]. The administration of neutralizing antibodies against CCL2 and CCL5 enhances the effectiveness of opioids [109]. Recent data emphasize that cytokines alter the effectiveness of opioids and the development of tolerance to their analgesic effects, which has been demonstrated in various animal models of pain [124,131,132,133,134,135,136]. Our research in CCI-exposed rats and mice showed that single, intrathecal and intraperitoneal administration of C021 enhanced the analgesic effect of morphine and buprenorphine [33,35]. We confirmed that the repeated intraperitoneal administration of morphine led to the development of opioid tolerance in mice after nerve injury [35]. Repeated administration of C021 alone for 12 days diminished nociceptive hypersensitivity. Although this effect was weaker in the few first days than morphine, it remained constant until the end of the experiment. Notably, the intraperitoneal administration of C021 with morphine or buprenorphine for 12 days significantly prolonged the analgesic effects of these opioids, and mice showed better locomotor activity on the RotaRod test.

Overall, our results indicate that targeting CCR4 and its ligands is a promising strategy to provide relief from neuropathic pain and may beneficially influence the analgesic effect of opioids, which represents a promising basis for the development of a more effective combined therapy for neuropathic pain.

## 5. CCR4 and Immunological Diseases

As previously mentioned, CCR4 is expressed by Th2 cells [63], regulatory T cells (Tregs) [137], mast cells [138], and skin-homing lymphocyte Ag–positive T cells [139]. Therefore, it plays an important role in inflammatory diseases, such as atopic dermatitis [140,141,142], asthma [54,143], and allergic airway inflammation [144], which are often associated with massive infiltration of Th2-type CD4+ T cells [145]. Studies using murine atopic dermatitis models showed that CCR4 deficiency or the use of a CCR4 antagonist ameliorated allergic responses. These results showed that CCR4 functioned in skin allergy inflammation by recruiting CCR4-expressing Th2 cells and Th17 cells [36,142]. There was also an increase in the expression of eosinophils, mast cells, Th2 cells, CCL17, and CCL22 in atopic dermatitis [36]. The CCR4/CCL17/CCL22 interaction is significant in the late phase of allergic airway inflammation, which was demonstrated using blocking antigens specific for CCL22 and CCL17 [52,66]. The blockade of CCL17 or CCL22 using neutralizing antibodies effectively reduced leukocyte recruitment to the lungs following allergen exposure [66,146]. Notably, CCL17 and CCL22 influence CCR4-expressing immune cells in the lungs and skin to evoke protective pulmonary responses to pathogens by recruiting Th2 and CD4+ T cells [39,137,147].

Encephalitis is a broad group of inflammatory diseases of the central nervous system. The causes of this condition may be an infection or an autoimmune disease. Experimental autoimmune encephalomyelitis (EAE) is a model that shares many characteristic features with multiple sclerosis (MS) [41,148]. The mechanism of EAE development involves the infiltration of mononuclear cells into the spinal cord, such as macrophages, dendritic, CD4+, and CD8+ T cells, which cause inflammation and demyelination [148,149]. An increase in CCL22 and CCR4 expression was demonstrated in the central nervous system in mice that develop chronic recurrent forms of autoimmune encephalitis, which demonstrates the involvement of CCL17/CCL22/CCR4 in the pathogenesis of this disease [148]. Experiments in animal models showed that CCR4 knockout mice were fully resistant to EAE and showed reduced neuritis and demyelination [148]. Increasing evidence from clinical trials indicates the involvement of the CCL17/CCL22/CCR4 axis in the pathogenesis of multiple sclerosis [41,50,150]. Cerebrospinal fluid levels of CCL22 and CCL17 are elevated in patients suffering from multiple sclerosis [41,50,151]. However, an increase in CCL22 levels was observed only in women [41].

CCR4 is also important in the pathogenesis of vitiligo, which is characterized by skin depigmentation. CCR4 and CCL17 were highly expressed in the skins of patients with vitiligo. The CCL17/CCR4 axis is also important for the onset of vitiligo in mice, and CCR4 neutralization reversed depigmentation in animals [152]. The levels of CCL17 and CCR4 were significantly upregulated in the pathogenesis of the oral lichen planus compared to the control patients [153].

The role of another CCR4 ligand, CCL2, in immunological diseases is primarily understood from its actions via CCR2. There is a direct association between the expression of hypoxia-inducible factor 1 and CCL2 during allergic lung inflammation in mice [154]. Mas receptor activation significantly attenuated CCL2-dependent macrophage recruitment in acute allergic airway inflammation via the JNK pathway [155].

Taken together, the interaction between CCR4, its ligands, and its modifications are important in the pathomechanism and treatment of immunological diseases.

## 6. CCR4 and Neoplastic Diseases

Adult T-cell leukemia/lymphoma (ATL) is caused by human T-lymphotrophic virus type 1 [38,101,102]. T-cell lines derived from patients with ATL are characterized by a high CCR4 expression at the mRNA and protein levels [37,102]. CCR4 is also expressed in the tumor cells of most ATL patients [37,102]. CCR4 is especially expressed on Treg lymphocytes and contributes to the influx of cells to the site of inflammation. Treg cells play an essential role in the maintenance of immune balance, but these cells interfere with the host’s antitumor immunity in malignant tumors and provide an environment for tumor growth [156,157]. Mogamulizumab was the first approved glycoengineered therapeutic antibody that targets CCR4. Clinical studies indicate that it recognizes the extracellular N-terminus of this receptor and provides beneficial effects in the treatment of ATL patients [101] It also demonstrated efficacy in the treatment of relapsed and refractory aggressive T-cell lymphomas [37,102]. An awareness of the importance of Treg cells in various cancers will allow for the rational design of more effective therapies. Reducing the number of Treg cells in cancer patients may be a promising strategy for immune enhancement and better immune therapy, even at the cost of autoimmunity, but with beneficial effects against the development of cancer cells [38,102].

CCR4 is also involved in the pathomechanism of hepatocellular carcinoma. Despite many studies, the basis of this disease is not clear. Frequent intrahepatic metastases lead to high mortality and poor prognosis of patients. Enhanced expression of CCL22 in the tumor tissues of patients with hepatocellular carcinoma was reported in some studies and was associated with accelerated tumor growth [158]. CCR4 promotes this malignancy and facilitates metastasis [159]. Notably, downregulated CCR4 consistently decreases the invasive capacity of hepatocellular carcinoma cells, and a CCR4 antagonist had antitumor effects in a murine model [160]. These findings suggest CCR4 is a potential new diagnostic and prognostic marker in hepatocellular carcinoma, and its targeting may be a new therapeutic strategy for blocking metastasis [159].

The expression of chemokine receptors (CXCR4, CCR7, and CCR10) was previously associated with breast cancer metastases [39]. Recent studies showed that CCR4 also promoted the growth of breast tumors in mice, and CCL17 overexpression enhanced the chemotactic response of neoplastic cells [39]. Notably, the pathomechanism of metastatic breast cancer involves primary tumor growth in mammary pads, which activates the expression of CCL17 and CCL22 in the lungs [39]. It is important to emphasize that Treg cell activation inhibits antitumor T-cell immune responses [161,162]. CCR4-mediated chemotaxis is not sufficient to produce metastasis because it requires CCR4-positive Treg cells, which suppress the cytotoxicity of NK cells that eliminate tumor cells [39,163]. The killing of CCR4-expressing cells via the delivery of CCL17-fused toxins or Treg depletion prevents lung metastasis [39]. Strategies that target CCR4-positive Treg cells may have significant benefits in the control of breast cancer metastasis by protecting adaptive immune responses [39,164].

An enhanced CCR4 expression is also correlated with the clinical stage and outcome of nonsmall cell lung cancer patients compared to controls [163]. A higher expression of the *CCR4* gene was confirmed in patients with lung adenocarcinoma [165]. The chemokines CCL17 and CCL22 regulate CCR4-expressing immune cells in the lungs to evoke protective pulmonary responses to pathogens.

CCR4 is also highly expressed in human renal cell carcinoma biopsies and plasma samples, which is associated with the extent of immune cell infiltration [40]. This enhancement is considered a poor survival prognosis for patients [166]. The levels of CCL17 and CCL22 are also changed in renal cancer tissue, and the CCL17/CCL22 ratio in plasma is associated with poor prognosis [40]. This finding is in agreement with studies of other solid tumors, where CCR4 is also highly expressed. Therefore, an anti-CCR4 antibody has antitumor activity. Although CCR4 inhibition does not reduce the proportion of infiltrating leukocytes, it alters the phenotype of myeloid cells and increases NK cells and Th1 cytokine levels, which potentially evokes antitumor activity [40]. The anti-CCR4 antibody, alone or in coadministration with other immune modulators, may be a potential treatment approach to cancer therapy.

The CCL2-CCR2 signaling axis plays a role in the promotion of pathological angiogenesis in neoplastic disease, and the survival and invasion of tumor cells, without taking CCR4 into account [167]. However, the latest studies by Ling and colleagues [168]. Showed that the CCL2/CCR4 axis, not CCL2/CCR2, induced the signaling cascade responsible for cell motility and metastasis in head and neck squamous cell carcinoma. Targeting CCR4 was also effective in disrupting CCL2-induced growth and metastasis without promoting cancer relapse [168]. Further research is undoubtedly necessary to evaluate the role of the CCL2/CCR4 axis in other cancers.

## 7. Future Perspectives and Treatment Strategies

The CCL2/CCL17/CCL22/CCR4 interaction is a promising target for the treatment and prevention of immune diseases, cancer, and neuropathic pain (Table 1). Therefore, the search for drugs that interact via CCR4 is a very interesting research area. Although the results of animal models are encouraging and show very promising results, many challenges must be resolved before CCR4-targeted therapies may be successfully developed for patients. Most compounds are in the preclinical phase of research. The expression of CCR4 on Th2 cells makes it a potential therapeutic target for allergic diseases. Due to the expression of CCR4 on Treg cells, the blockade of CCR4 may also be beneficial in enhancing the effectiveness of anticancer vaccines. As mentioned earlier, studies have already identified several small molecule antagonists of the CCR4 receptor and have tested and evaluated these agents in animal models of allergic diseases. Based on the research results, we conclude that CCR4 and its ligands are involved in the pathogenesis of many diseases of various etiologies. Promising studies offer hope for a new strategy for effective polytherapy in patients suffering from neuropathic pain, several immunological diseases, and cancer.

**Table 1:** Summary of the involvement of CCL2, CCL17, and CCL22 in different health problems in the cases of pain, immunity, and tumor studies.

LIGAND	PAIN	IMMUNITY	TUMORS
**CCL17** **T**hymus and **A**ctivation-**R**egulated **C**hemokine **(TARC)** **CELL TYPES ** neurons, lymphocytes, basophils, mononuclear cells, dendritic cells	**CLINICAL STUDY**		
	fibromyalgia—clinical trials [71]	asthma [143] dermatitis [140] autoimmune diseases [57,59,60,61] atherosclerosis [61]	breast cancer [169] hepatocellural carcinoma [170] colon cancer– clinical trials [171]
	**EXPERIMENTAL STUDY**		
	neuropathic pain [33,34,35] inflammatory pain [172]	asthma [173]	pituitary adenoma [174] glioblastoma [175] gastric cancer [176]
**CCL22** **M**acrophage-**D**erived **C**hemokine-**(MDC)** **CELL TYPES** lymphocytes, basophils, NK, mononuclear cells, dendritic cells	**CLINICAL STUDY**		
	fibromyalgia [71]	atopic dermatitis [137] allergy [64] asthma [143,177] dermatitis [178] pneumonia [179]	breast cancer [169] hepatocellural carcinoma [170] colon cancer [171] gastric cancer [176]
	**EXPERIMENTAL STUDY**		
	neuropathic pain [33,34,35]	autoimmune encephalomyelitis [106]	melanoma [180] t-cell lymphoma [181] hepatocellular carcinoma [182] bladder cancer [183]
**CCL2** **M**onocyte **C**hemoattractant **P**rotein-**1 (MCP-1)** **CELL TYPES** T lymphocytes, NK cells monocytes/macrophages, endothelial/epithelial cells, fibroblasts, smooth muscle, astroglia and microglia	**CLINICAL STUDY**		
	lumbar disk herniation [184] traumatic spinal cord injury [185]	multiple sclerosis [186] tuberculosis [187] myocardial infarction [188] aids [189] multiple sclerosis [190] nephropathy [191] inflammatory bowel disease [192] allergic asthma [193] rheumatoid arthritis [194]	colorectal cancer [195] ovarian cancer [195] esophagus cancer [195] pancreatic cancer [195] breast cancer [195]
	**EXPERIMENTAL STUDY**		
	neuropathic pain [15,16,109,119] arthritis bone cancer pain [196]	insulin resistance [197] asthma [198] autoimmune encephalomyelitis [199]	meningioma [200] colon cancer [201] carcinomas [202] melanoma [203] bladder cancer [204]

## Data Availability

Not applicable.

## References

[1] Zlotnik A., Yoshie O. (2012). The Chemokine Superfamily Revisited. Immunity.

[2] Zhang Z.-J., Jiang B.-C., Gao Y.-J. (2017). Chemokines in neuron–glial cell interaction and pathogenesis of neuropathic pain. Cell. Mol. Life Sci..

[3] Charo I.F., Ransohoff R.M. (2006). The Many Roles of Chemokines and Chemokine Receptors in Inflammation. N. Engl. J. Med..

[4] Gao Y.-J., Ji R.-R. (2010). Chemokines, neuronal–glial interactions, and central processing of neuropathic pain. Pharmacol. Ther..

[5] Kwiatkowski K., Mika J. (2014). Chemokines under neuropathic pain. Ból.

[6] Cartier L., Hartley O., Dubois-Dauphin M., Krause K.-H. (2005). Chemokine receptors in the central nervous system: Role in brain inflammation and neurodegenerative diseases. Brain Res. Rev..

[7] Murdoch C., Finn A. (2000). Chemokine receptors and their role in inflammation and infectious diseases. Blood.

[8] Gao Y.-J., Ji R.-R. (2010). Targeting astrocyte signaling for chronic pain. Neurotherapeutics.

[9] Piotrowska A., Rojewska E., Pawlik K., Kreiner G., Ciechanowska A., Makuch W., Nalepa I., Mika J. (2019). Pharmacological Blockade of Spinal CXCL3/CXCR2 Signaling by NVP CXCR2 20, a Selective CXCR2 Antagonist, Reduces Neuropathic Pain Following Peripheral Nerve Injury. Front. Immunol..

[10] Sorensen T.L., Ransohoff R.M., Strieter R.M., Sellebjerg F. (2004). Chemokine CCL2 and chemokine receptor CCR2 in early active multiple sclerosis. Eur. J. Neurol..

[11] Mines M., Ding Y., Fan G.-H. (2007). The many roles of chemokine receptors in neurodegenerative disorders: Emerging new therapeutical strategies. Curr. Med. Chem..

[12] Ransohoff R.M. (2002). The Chemokine System in Neuroinflammation: An Update. J. Infect. Dis..

[13] Pawlik K., Piotrowska A., Kwiatkowski K., Ciapała K., Popiolek-Barczyk K., Makuch W., Mika J. (2020). The blockade of CC chemokine receptor type 1 influences the level of nociceptive factors and enhances opioid analgesic potency in a rat model of neuropathic pain. Immunology.

[14] Rojewska E., Zychowska M., Piotrowska A., Kreiner G., Nalepa I., Mika J. (2018). Involvement of Macrophage Inflammatory Protein-1 Family Members in the Development of Diabetic Neuropathy and Their Contribution to Effectiveness of Morphine. Front. Immunol..

[15] Kwiatkowski K., Piotrowska A., Rojewska E., Makuch W., Mika J. (2017). The RS504393 Influences the Level of Nociceptive Factors and Enhances Opioid Analgesic Potency in Neuropathic Rats. J. Neuroimmune Pharmacol..

[16] Piotrowska A., Kwiatkowski K., Rojewska E., Slusarczyk J., Makuch W., Basta-Kaim A., Przewlocka B., Mika J. (2016). Direct and indirect pharmacological modulation of CCL2/CCR2 pathway results in attenuation of neuropathic pain—In vivo and in vitro evidence. J. Neuroimmunol..

[17] Kwiatkowski K., Piotrowska A., Rojewska E., Makuch W., Jurga A., Slusarczyk J., Trojan E., Basta-Kaim A., Mika J. (2016). Beneficial properties of maraviroc on neuropathic pain development and opioid effectiveness in rats. Prog. Neuro-Psychopharmacol. Biol. Psychiatry.

[18] Piotrowska A., Kwiatkowski K., Rojewska E., Makuch W., Mika J. (2016). Maraviroc reduces neuropathic pain through polarization of microglia and astroglia—Evidence from in vivo and in vitro studies. Neuropharmacology.

[19] Zychowska M., Rojewska E., Piotrowska A., Kreiner G., Nalepa I., Mika J. (2017). Spinal CCL1/CCR8 signaling interplay as a potential therapeutic target—Evidence from a mouse diabetic neuropathy model. Int. Immunopharmacol..

[20] Piotrowska A., Rojewska E., Pawlik K., Kreiner G., Ciechanowska A., Makuch W., Mika J. (2018). Dataset of (±)-NBI-74330 (CXCR3 antagonist) influence on chemokines under neuropathic pain. Data Brief.

[21] Bhangoo S.K., Ren D., Miller R.J., Chan D., Ripsch M.S., Weiss C., McGinnis C., White F.A. (2007). CXCR4 chemokine receptor signaling mediates pain hypersensitivity in association with antiretroviral toxic neuropathy. Brain Behav. Immun..

[22] Dubový P., Klusáková I., Svizenska I., Brazda V. (2010). Spatio-temporal changes of SDF1 and its CXCR4 receptor in the dorsal root ganglia following unilateral sciatic nerve injury as a model of neuropathic pain. Histochem. Cell Biol..

[23] Zychowska M., Rojewska E., Piotrowska A., Kreiner G., Mika J. (2016). Microglial Inhibition Influences XCL1/XCR1 Expression and Causes Analgesic Effects in a Mouse Model of Diabetic Neuropathy. Anesthesiology.

[24] Tokuyama H., Ueha S., Kurachi M., Matsushima K., Moriyasu F., Blumberg R.S., Kakimi K. (2005). The simultaneous blockade of chemokine receptors CCR2, CCR5 and CXCR3 by a non-peptide chemokine receptor antagonist protects mice from dextran sodium sulfate-mediated colitis. Int. Immunol..

[25] Haringman J.J., Kraan M.C., Smeets T.J.M., Zwinderman K.H., Tak P.P. (2003). Chemokine blockade and chronic inflammatory disease: Proof of concept in patients with rheumatoid arthritis. Ann. Rheum. Dis..

[26] Liu J. (2010). CC Chemokine Receptor Small Molecule Antagonists in the Treatment of Rheumatoid Arthritis and Other Diseases: A Current View. Curr. Top. Med. Chem..

[27] Elsner J., Escher S.E., Forssmann U. (2004). Chemokine receptor antagonists: A novel therapeutic approach in allergic diseases. Allergy.

[28] Vangelista L., Vento S. (2018). The Expanding Therapeutic Perspective of CCR5 Blockade. Front. Immunol..

[29] Miao M., De Clercq E., Li G. (2020). Clinical significance of chemokine receptor antagonists. Expert Opin. Drug Metab. Toxicol..

[30] Xia J., Sun S., Jotte M.R., Uy G.L., Bohana-Kashtan O., Sorani E., Vainstein A., Peled A., Link D.C. (2019). CXCR4 Blockade By BL-8040 in T Cell Acute Lymphoblastic Leukemia Decreases Mitochondrial Mass and Induces Non-Apoptotic Cell Death. Blood.

[31] Dhaiban S., Al-Ani M., Elemam N.M., A Maghazachi A. (2020). Targeting Chemokines and Chemokine Receptors in Multiple Sclerosis and Experimental Autoimmune Encephalomyelitis. J. Inflamm. Res..

[32] Zhang Y., Wu Y., Qi H., Xiao J., Gong H., Xu E., Li S., Ma D., Wang Y., Li W. (2017). A new antagonist for CCR4 attenuates allergic lung inflammation in a mouse model of asthma. Sci. Rep..

[33] Bogacka J., Popiolek-Barczyk K., Pawlik K., Ciechanowska A., Makuch W., Rojewska E., Dobrogowski J., Przeklasa-Muszynska A., Mika J. (2020). CCR4 antagonist (C021) influences the level of nociceptive factors and enhances the analgesic potency of morphine in a rat model of neuropathic pain. Eur. J. Pharmacol..

[34] Bogacka J., Ciapała K., Pawlik K., Dobrogowski J., Przeklasa-Muszynska A., Mika J. (2020). Blockade of CCR4 Diminishes Hypersensitivity and Enhances Opioid Analgesia—Evidence from a Mouse Model of Diabetic Neuropathy. Neuroscience.

[35] Bogacka J., Ciapała K., Pawlik K., Kwiatkowski K., Dobrogowski J., Przeklasa-Muszynska A., Mika J. (2020). CCR4 Antagonist (C021) Administration Diminishes Hypersensitivity and Enhances the Analgesic Potency of Morphine and Buprenorphine in a Mouse Model of Neuropathic Pain. Front. Immunol..

[36] Matsuo K., Nagakubo D., Komori Y., Fujisato S., Takeda N., Kitamatsu M., Nishiwaki K., Quan Y.-S., Kamiyama F., Oiso N. (2018). CCR4 Is Critically Involved in Skin Allergic Inflammation of BALB/c Mice. J. Investig. Dermatol..

[37] Yoshie O., Matsushima K. (2014). CCR4 and its ligands: From bench to bedside. Int. Immunol..

[38] Ishida T., Ito A., Sato F., Kusumoto S., Iida S., Inagaki H., Morita A., Akinaga S., Ueda R. (2013). Stevens-Johnson Syndrome associated with mogamulizumab treatment of adult T-cell leukemia/lymphoma. Cancer Sci..

[39] Olkhanud P.B., Baatar D., Bodogai M., Hakim F., Gress R., Anderson R.L., Deng J., Xu M., Briest S., Biragyn A. (2009). Breast Cancer Lung Metastasis Requires Expression of Chemokine Receptor CCR4 and Regulatory T Cells. Cancer Res..

[40] Berlato C., Khan M.N., Schioppa T., Thompson R., Maniati E., Montfort A., Jangani M., Canosa M., Kulbe H., Hagemann U.B. (2017). A CCR4 antagonist reverses the tumor-promoting microenvironment of renal cancer. J. Clin. Investig..

[41] Scheu S., Ali S., Ruland C., Arolt V., Alferink J. (2017). The C-C Chemokines CCL17 and CCL22 and Their Receptor CCR4 in CNS Autoimmunity. Int. J. Mol. Sci..

[42] McMillin M., Frampton G., Thompson M., Galindo C., Standeford H., Whittington E., Alpini G., DeMorrow S. (2014). Neuronal CCL2 is upregulated during hepatic encephalopathy and contributes to microglia activation and neurological decline. J. Neuroinflammation.

[43] Salanga C., Handel T. (2011). Chemokine oligomerization and interactions with receptors and glycosaminoglycans: The role of structural dynamics in function. Exp. Cell Res..

[44] Meucci O., Fatatis A., Simen A.A., Bushell T.J., Gray P.W., Miller R.J. (1998). Chemokines regulate hippocampal neuronal signaling and gp120 neurotoxicity. Proc. Natl. Acad. Sci. USA.

[45] Flynn G., Maru S., Loughlin J., A Romero I., Male D. (2003). Regulation of chemokine receptor expression in human microglia and astrocytes. J. Neuroimmunol..

[46] Kufareva I., Gustavsson M., Zheng Y., Stephens B.S., Handel T.M. (2017). What Do Structures Tell Us About Chemokine Receptor Function and Antagonism?. Annu. Rev. Biophys..

[47] Scholten D., Canals M., Maussang D., Roumen L., Smit M., Wijtmans M., de Graaf C., Vischer H., Leurs R. (2012). Pharmacological modulation of chemokine receptor function. Br. J. Cereb. Blood Flow Metab..

[48] Power C.A., Meyer A., Nemeth K., Bacon K.B., Hoogewerf A.J., Proudfoot A.E., Wells T.N. (1995). Molecular Cloning and Functional Expression of a Novel CC Chemokine Receptor cDNA from a Human Basophilic Cell Line. J. Biol. Chem..

[49] Oh S.B., Tran P.B., Gillard S.E., Hurley R., Hammond D., Miller R.J. (2001). Chemokines and Glycoprotein120 Produce Pain Hypersensitivity by Directly Exciting Primary Nociceptive Neurons. J. Neurosci..

[50] Jafarzadeh A., Arabi Z., Ahangar-Parvin R., Mohammadi-Kordkhayli M., Nemati M. (2017). Ginger Extract Modulates the Expression of Chemokines CCL20 and CCL22 and Their Receptors (CCR6 and CCR4) in the Central Nervous System of Mice with Experimental Autoimmune Encephalomyelitis. Drug Res..

[51] Bajetto A., Bonavia R., Barbero S., Schettini G. (2002). Characterization of chemokines and their receptors in the central nervous system: Physiopathological implications. J. Neurochem..

[52] Viney J.M., Andrew D.P., Phillips R.M., Meiser A., Patel P., Lennartz-Walker M., Cousins D.J., Barton N.P., Hall D.A., Pease J.E. (2014). Distinct Conformations of the Chemokine Receptor CCR4 with Implications for Its Targeting in Allergy. J. Immunol..

[53] Moriguchi K., Miyamoto K., Tanaka N., Ueno R., Nakayama T., Yoshie O., Kusunoki S. (2015). C-C chemokine receptor type 4 antagonist Compound 22 ameliorates experimental autoimmune encephalomyelitis. J. Neuroimmunol..

[54] Pilette C., Francis J., Till S., Durham S. (2004). CCR4 ligands are up-regulated in the airways of atopic asthmatics after segmental allergen challenge. Eur. Respir. J..

[55] Kiguchi N., Ding H., Peters C.M., Kock N.D., Kishioka S., Cline J.M., Wagner J.D., Ko M.-C. (2017). Altered expression of glial markers, chemokines, and opioid receptors in the spinal cord of type 2 diabetic monkeys. Biochim. Biophys. Acta (BBA)-Mol. Basis Dis..

[56] Nomiyama H., Imaib T., Kusudac J., Miuraa R., Callen D., Yoshie O. (1997). Assignment of the Human CC Chemokine Gene TARC (SCYA17) to Chromosome 16q13. Genomics.

[57] Alferink J., Lieberam I., Reindl W., Behrens A., Weiss S., Hüser N., Gerauer K., Ross R., Reske-Kunz A.B., Ahmad-Nejad P. (2003). Compartmentalized Production of CCL17 In Vivo: Strong inducibility in peripheral dendritic cells contrasts selective absence from the spleen. J. Exp. Med..

[58] Fülle L., Offermann N., Hansen J.N., Breithausen B., Erazo A.B., Schanz O., Radau L., Gondorf F., Knöpper K., Alferink J. (2018). CCL17 exerts a neuroimmune modulatory function and is expressed in hippocampal neurons. Glia.

[59] Heiseke A.F., Faul A.C., Lehr H., Förster I., Schmid R.M., Krug A.B., Reindl W. (2012). CCL17 Promotes Intestinal Inflammation in Mice and Counteracts Regulatory T Cell–Mediated Protection From Colitis. Gastroenterology.

[60] Stutte S., Quast T., Gerbitzki N., Savinko T., Novak N., Reifenberger J., Homey B., Kolanus W., Alenius H., Förster I. (2010). Requirement of CCL17 for CCR7- and CXCR4-dependent migration of cutaneous dendritic cells. Proc. Natl. Acad. Sci. USA.

[61] Weber C., Meiler S., Döring Y., Koch M., Drechsler M., Megens R., Rowinska Z., Bidzhekov K., Fecher C., Ribechini E. (2011). CCL17-expressing dendritic cells drive atherosclerosis by restraining regulatory T cell homeostasis in mice. J. Clin. Investig..

[62] Imai T., Baba M., Nishimura M., Kakizaki M., Takagi S., Yoshie O. (1997). The T Cell-directed CC Chemokine TARC Is a Highly Specific Biological Ligand for CC Chemokine Receptor 4. J. Biol. Chem..

[63] Bonecchi R., Galliera E., Borroni E.M., Corsi M.M., Locati M., Mantovani A. (2009). Chemokines and chemokine receptors: An overview. Front. Biosci..

[64] Yamashita U., Kuroda E. (2002). Regulation of Macrophage-Derived Chemokine (MDC/CCL22) Production. Crit. Rev. Immunol..

[65] Montane J., Bischoff L., Soukhatcheva G., Dai D.L., Hardenberg G., Levings M., Orban P.C., Kieffer T.J., Tan R., Verchere C.B. (2011). Prevention of murine autoimmune diabetes by CCL22-mediated Treg recruitment to the pancreatic islets. J. Clin. Investig..

[66] Kawasaki Y., Xu Z.-Z., Wang X., Park J.Y., Zhuang Z.-Y., Tan P.-H., Gao Y.-J., Roy K., Corfas G., Lo E.H. (2008). Distinct roles of matrix metalloproteases in the early- and late-phase development of neuropathic pain. Nat. Med..

[67] Mikhak Z., Fukui M., Farsidjani A., Medoff B., Tager A.M., Luster A.D. (2009). Contribution of CCR4 and CCR8 to antigen-specific TH2 cell trafficking in allergic pulmonary inflammation. J. Allergy Clin. Immunol..

[68] Li Y.-Q., Liu F.-F., Zhang X.-M., Guo X.-J., Ren M.-J., Fu L. (2013). Tumor Secretion of CCL22 Activates Intratumoral Treg Infiltration and Is Independent Prognostic Predictor of Breast Cancer. PLoS ONE.

[69] Martinenaite E., Ahmad S.M., Hansen M., Met Ö., Westergaard M.W., Larsen S.K., Klausen T.W., Donia M., Svane I.M., Andersen M.H. (2016). CCL22-specific T Cells: Modulating the immunosuppressive tumor microenvironment. OncoImmunology.

[70] Imai T., Chantry D., Raport C.J., Wood C.L., Nishimura M., Godiska R., Yoshie O., Gray P.W. (1998). Macrophage-derived Chemokine Is a Functional Ligand for the CC Chemokine Receptor 4. J. Biol. Chem..

[71] García J.J., Cidoncha A., E Bote M., Hinchado M.D., Ortega E. (2014). Altered profile of chemokines in fibromyalgia patients. Ann. Clin. Biochem..

[72] Frossard J.L., Lenglet S., Montecucco F., Steffens S., Galan K., Pelli G., Spahr L., Mach F., Hadengue A. (2011). Role of CCL-2, CCR-2 and CCR-4 in cerulein-induced acute pancreatitis and pancreatitis-associated lung injury. J. Clin. Pathol..

[73] Xiong W., Tan J., Guo Y., Chen S., Fan L., Li Y. (2020). Notch3 Knockout Suppresses Mouse Mammary Gland Development and Inhibits the Proliferation of 4T1 Murine Mammary Carcinoma Cells via CCL2/CCR4 Axis. Front. Cell Dev. Biol..

[74] Yoshimura T., A Robinson E., Tanaka S., Appella E., Leonard E.J. (1989). Purification and amino acid analysis of two human monocyte chemoattractants produced by phytohemagglutinin-stimulated human blood mononuclear leukocytes. J. Immunol..

[75] Barna B.P., Pettay J., Barnett G.H., Zhou P., Iwasaki K., Estes M.L. (1994). Regulation of monocyte chemoattractant protein-1 expression in adult human non-neoplastic astrocytes is sensitive to tumor necrosis factor (TNF) or antibody to the 55-kDa TNF receptor. J. Neuroimmunol..

[76] Brown Z., Strieter R.M., Neild G.H., Thompson R.C., Kunkel S.L., Westwick J. (1992). IL-1 receptor antagonist inhibits monocyte chemotactic peptide 1 generation by human mesangial cells. Kidney Int..

[77] A Begley L., Kasina S., Mehra R., Adsule S., Admon A.J., Lonigro R.J., Chinnaiyan A.M., A Macoska J. (2008). CXCL5 Promotes Prostate Cancer Progression. Neoplasia.

[78] Singh R.K., Lokeshwar B.L. (2009). Depletion of intrinsic expression of Interleukin-8 in prostate cancer cells causes cell cycle arrest, spontaneous apoptosis and increases the efficacy of chemotherapeutic drugs. Mol. Cancer.

[79] Dutta P., Sarkissyan M., Paico K., Wu Y., Vadgama J.V. (2018). MCP-1 is overexpressed in triple-negative breast cancers and drives cancer invasiveness and metastasis. Breast Cancer Res. Treat..

[80] Hayashida K., Nanki T., Girschick H., Yavuz S., Ochi T., E Lipsky P. (2001). Synovial stromal cells from rheumatoid arthritis patients attract monocytes by producing MCP-1 and IL-8. Arthritis Res..

[81] Kusano K.F., Nakamura K., Kusano H., Nishii N., Banba K., Ikeda T., Hashimoto K., Yamamoto M., Fujio H., Miura A. (2004). Significance of the Level of Monocyte Chemoattractant Protein-1 in Human Atherosclerosis-Assessment in Chronic Hemodialysis Patients. Circ. J..

[82] Sartipy P., Loskutoff D.J. (2003). Monocyte chemoattractant protein 1 in obesity and insulin resistance. Proc. Natl. Acad. Sci. USA.

[83] Van Steenwinckel J., LE Goazigo A.R., Pommier B., Mauborgne A., Dansereau M.-A., Kitabgi P., Sarret P., Pohl M., Parsadaniantz S.M. (2011). CCL2 Released from Neuronal Synaptic Vesicles in the Spinal Cord Is a Major Mediator of Local Inflammation and Pain after Peripheral Nerve Injury. J. Neurosci..

[84] Bose S., Cho J. (2013). Role of chemokine CCL2 and its receptor CCR2 in neurodegenerative diseases. Arch. Pharmacal Res..

[85] Ren F., Jiao H., Cai H. (2015). Analgesic Effect of Intrathecal Administration of Chemokine Receptor CCR2 Antagonist is Related to Change in Spinal NR2B, nNOS, and SIGIRR Expression in Rat with Bone Cancer Pain. Cell Biophys..

[86] Jin D., Yang J.-P., Hu J.-H., Wang L.-N., Zuo J.-L. (2015). MCP-1 stimulates spinal microglia via PI3K/Akt pathway in bone cancer pain. Brain Res..

[87] Nikodemova M., Duncan I.D., Watters J.J. (2006). Minocycline exerts inhibitory effects on multiple mitogen-activated protein kinases and IκBα degradation in a stimulus-specific manner in microglia. J. Neurochem..

[88] Purandare A., Somerville J. (2006). Antagonists of CCR4 as Immunomodulatory Agents. Curr. Top. Med. Chem..

[89] Purandare A.V., Gao A., Wan H., Somerville J., Burke C., Seachord C., Vaccaro W., Wityak J., Poss M.A. (2005). Identification of chemokine receptor CCR4 antagonist. Bioorg. Med. Chem. Lett..

[90] Andrews G., Jones C., Wreggett K.A. (2008). An Intracellular Allosteric Site for a Specific Class of Antagonists of the CC Chemokine G Protein-Coupled Receptors CCR4 and CCR5. Mol. Pharmacol..

[91] Banfield G., Watanabe H., Scadding G., Jacobson M.R., Till S.J., Hall D.A., Robinson D.S., Lloyd C.M., Nouri-Aria K.T., Durham S.R. (2010). CC Chemokine Receptor 4 (CCR4) in human allergen-induced late nasal responses. Allergy.

[92] Burdi D.F., Chi S., Mattia K., Harrington C., Shi Z., Chen S., Jacutin-Porte S., Bennett R., Carson K., Yin W. (2007). Small molecule antagonists of the CC chemokine receptor 4 (CCR4). Bioorg. Med. Chem. Lett..

[93] Cahn A., Hodgson S., Wilson R., Robertson J., Watson J., Beerahee M., Hughes S.C., Young G., Graves R., Hall D. (2013). Safety, tolerability, pharmacokinetics and pharmacodynamics of GSK2239633, a CC-chemokine receptor 4 antagonist, in healthy male subjects: Results from an open-label and from a randomised study. BMC Pharmacol. Toxicol..

[94] Kuhn C.F., Bazin M., Philippe L., Zhang J., Tylaska L., Miret J., Bauer P.H. (2007). Bipiperidinyl Carboxylic Acid Amides as Potent, Selective, and Functionally Active CCR4 Antagonists. Chem. Biol. Drug Des..

[95] Solari R., Pease J.E. (2015). Targeting chemokine receptors in disease—A case study of CCR4. Eur. J. Pharmacol..

[96] Yokoyama K., Ishikawa N., Igarashi S., Kawano N., Masuda N., Hamaguchi W., Yamasaki S., Koganemaru Y., Hattori K., Miyazaki T. (2009). Potent and orally bioavailable CCR4 antagonists: Synthesis and structure–activity relationship study of 2-aminoquinazolines. Bioorg. Med. Chem..

[97] Yokoyama K., Ishikawa N., Igarashi S., Kawano N., Masuda N., Hattori K., Miyazaki T., Ogino S.-I., Orita M., Matsumoto Y. (2008). Potent CCR4 antagonists: Synthesis, evaluation, and docking study of 2,4-diaminoquinazolines. Bioorg. Med. Chem..

[98] Kindon N., Andrews G., Baxter A., Cheshire D., Hemsley P., Johnson T., Liu Y.-Z., McGinnity D., McHale M., Mete A. (2017). Discovery of AZD-2098 and AZD-1678, Two Potent and Bioavailable CCR4 Receptor Antagonists. ACS Med. Chem. Lett..

[99] Suzuki Y., Saito M., Ishii T., Urakawa I., Matsumoto A., Masaki A., Ito A., Kusumoto S., Suzuki S., Hiura M. (2019). Mogamulizumab Treatment Elicits Autoantibodies Attacking the Skin in Patients with Adult T-Cell Leukemia-Lymphoma. Clin. Cancer Res..

[100] Doi T., Muro K., Ishii H., Kato T., Tsushima T., Takenoyama M., Oizumi S., Gemmoto K., Suna H., Enokitani K. (2019). A phase 1 study of the anti-CC chemokine receptor 4 antibody, mogamulizumab, in combination with nivolumab in patients with advanced or metastatic solid tumors. Clin. Cancer Res..

[101] Remer M., Al-Shamkhani A., Glennie M., Johnson P. (2014). Mogamulizumab and the treatment of CCR4-positive T-cell lymphomas. Immunotherapy.

[102] Ishida T., Joh T., Uike N., Yamamoto K., Utsunomiya A., Yoshida S., Saburi Y., Miyamoto T., Takemoto S., Suzushima H. (2012). Defucosylated Anti-CCR4 Monoclonal Antibody (KW-0761) for Relapsed Adult T-Cell Leukemia-Lymphoma: A Multicenter Phase II Study. J. Clin. Oncol..

[103] Abboud D., Daubeuf F., Do Q.T., Utard V., Villa P., Haiech J., Bonnet D., Hibert M., Bernard P., Galzi J.-L. (2015). A strategy to discover decoy chemokine ligands with an anti-inflammatory activity. Sci. Rep..

[104] Teng K.-Y., Han J., Zhang X., Hsu S.-H., He S., Wani N.A., Barajas J.M., Snyder L.A., Frankel W.L., Caligiuri M.A. (2017). Blocking the CCL2–CCR2 Axis Using CCL2-Neutralizing Antibody Is an Effective Therapy for Hepatocellular Cancer in a Mouse Model. Mol. Cancer Ther..

[105] Liu L.-B., Xie F., Chang K.-K., Shang W.-Q., Meng Y.-H., Yu J.-J., Li H., Sun Q., Yuan M.-M., Jin L.-P. (2015). Chemokine CCL17 induced by hypoxia promotes the proliferation of cervical cancer cell. Am. J. Cancer Res..

[106] Kwiatkowski K., Popiolek-Barczyk K., Piotrowska A., Rojewska E., Ciapała K., Makuch W., Mika J. (2019). Chemokines CCL2 and CCL7, but not CCL12, play a significant role in the development of pain-related behavior and opioid-induced analgesia. Cytokine.

[107] Fang W.B., Yao M., Brummer G., Acevedo D., Alhakamy N., Berkland C., Cheng N. (2016). Targeted gene silencing of CCL2 inhibits triple negative breast cancer progression by blocking cancer stem cell renewal and M2 macrophage recruitment. Oncotarget.

[108] Zhu X., Fujita M., Snyder L.A., Okada H. (2011). Systemic delivery of neutralizing antibody targeting CCL2 for glioma therapy. J. Neuro-Oncol..

[109] Lee M.-C., Saleh R., Achuthan A., Fleetwood A.J., Förster I., Hamilton J.A., Cook A.D. (2018). CCL17 blockade as a therapy for osteoarthritis pain and disease. Arthritis Res. Ther..

[110] Lee K.-C., Prasad V., Achuthan A., Fleetwood A., Hamilton J., Cook A. (2020). Targeting GM-CSF for collagenase-induced osteoarthritis pain and disease in mice. Osteoarthr. Cartil..

[111] Dogan R.-N.E., Long N., Forde E., Dennis K., Kohm A.P., Miller S.D., Karpus W.J. (2010). CCL22 regulates experimental autoimmune encephalomyelitis by controlling inflammatory macrophage accumulation and effector function. J. Leukoc. Biol..

[112] Austin P.J., Moalem-Taylor G. (2010). The neuro-immune balance in neuropathic pain: Involvement of inflammatory immune cells, immune-like glial cells and cytokines. J. Neuroimmunol..

[113] Raghavendra V., Rutkowski M.D., DeLeo J.A. (2002). The Role of Spinal Neuroimmune Activation in Morphine Tolerance/Hyperalgesia in Neuropathic and Sham-Operated Rats. J. Neurosci..

[114] Ransohoff R.M. (2009). Chemokines and Chemokine Receptors: Standing at the Crossroads of Immunobiology and Neurobiology. Immunity.

[115] Mika J., Zychowska M., Popiolek-Barczyk K., Rojewska E., Przewlocka B. (2013). Importance of glial activation in neuropathic pain. Eur. J. Pharmacol..

[116] Dansereau M.-A., Gosselin R.-D., Pohl M., Pommier B., Mechighel P., Mauborgne A., Rostene W., Kitabgi P., Beaudet N., Sarret P. (2008). Spinal CCL2 pronociceptive action is no longer effective in CCR2 receptor antagonist-treated rats. J. Neurochem..

[117] Pfützner J., Hellhammer J., Musholt P., Pfützner A.H., Böhnke J., Hero T., Amann-Zalan I., Ganz M., Forst T., Pfützner A. (2011). Evaluation of Dexterity in Insulin-Treated Patients with Type 1 and Type 2 Diabetes Mellitus. J. Diabetes Sci. Technol..

[118] Mueller M.J., Minor S.D., A Sahrmann S., A Schaaf J., Strube M.J. (1994). Differences in the Gait Characteristics of Patients With Diabetes and Peripheral Neuropathy Compared With Age-Matched Controls. Phys. Ther..

[119] Kwiatkowski K., Ciapała K., Rojewska E., Makuch W., Mika J. (2020). Comparison of the beneficial effects of RS504393, maraviroc and cenicriviroc on neuropathic pain-related symptoms in rodents: Behavioral and biochemical analyses. Int. Immunopharmacol..

[120] Popiolek-Barczyk K., Makuch W., Rojewska E., Pilat D., Mika J. (2014). Inhibition of intracellular signaling pathways NF-κB and MEK1/2 attenuates neuropathic pain development and enhances morphine analgesia. Pharmacol. Rep..

[121] Hung A.L., Lim M., Doshi T.L. (2017). Targeting cytokines for treatment of neuropathic pain. Scand. J. Pain.

[122] Colloca L., Ludman T., Bouhassira D., Baron R., Dickenson A.H., Yarnitsky D., Freeman R., Truini A., Attal N., Finnerup N. (2017). Neuropathic pain. Nat. Rev. Dis. Primers.

[123] Zychowska M., Rojewska E., Kreiner G., Nalepa I., Przewlocka B., Mika J. (2013). Minocycline influences the anti-inflammatory interleukins and enhances the effectiveness of morphine under mice diabetic neuropathy. J. Neuroimmunol..

[124] Mika J., Osikowicz M., Rojewska E., Korostynski M., Wawrzczak-Bargiela A., Przewlocki R., Przewlocka B. (2009). Differential activation of spinal microglial and astroglial cells in a mouse model of peripheral neuropathic pain. Eur. J. Pharmacol..

[125] Cavalli E., Mammana S., Nicoletti F., Bramanti P., Mazzon E. (2019). The neuropathic pain: An overview of the current treatment and future therapeutic approaches. Int. J. Immunopathol. Pharmacol..

[126] Colvin L.A., Bull F., Hales T.G. (2019). Perioperative opioid analgesia—when is enough too much? A review of opioid-induced tolerance and hyperalgesia. Lancet.

[127] Lin C.-P., Lu D.-H. (2018). Role of Neuroinflammation in Opioid Tolerance: Translational Evidence from Human-to-Rodent Studies. Advances in Pain Research: Mechanisms and Modulation of Chronic Pain.

[128] Mika J., Wawrzczak-Bargiela A., Osikowicz M., Makuch W., Przewlocka B. (2009). Attenuation of morphine tolerance by minocycline and pentoxifylline in naive and neuropathic mice. Brain Behav. Immun..

[129] Pilat D., Piotrowska A., Rojewska E., Jurga A., Ślusarczyk J., Makuch W., Basta-Kaim A., Przewlocka B., Mika J. (2016). Blockade of IL-18 signaling diminished neuropathic pain and enhanced the efficacy of morphine and buprenorphine. Mol. Cell. Neurosci..

[130] Pilat D., Rojewska E., Jurga A.M., Piotrowska A., Makuch W., Przewlocka B., Mika J. (2015). IL-1 receptor antagonist improves morphine and buprenorphine efficacy in a rat neuropathic pain model. Eur. J. Pharmacol..

[131] Mika J. (2008). Review paper The opioid systems and the role of glial cells in the effects of opioids The opioid systems. Adv. Pall. Med..

[132] Liu Z., Song Z., Guo S., He J., Wang S., Zhu J., Yang H., Liu J. (2019). CXCL12/CXCR4 signaling contributes to neuropathic pain via central sensitization mechanisms in a rat spinal nerve ligation model. CNS Neurosci. Ther..

[133] Pan Y., Sun X., Jiang L., Hu L., Kong H., Han Y., Qian C., Song C., Qian Y., Liu W. (2016). Metformin reduces morphine tolerance by inhibiting microglial-mediated neuroinflammation. J. Neuroinflamm..

[134] Zhang L., Yu M., Deng J., Lv X., Liu J., Xiao Y., Yang W., Zhang Y., Li C. (2015). Chemokine Signaling Pathway Involved in CCL2 Expression in Patients with Rheumatoid Arthritis. Yonsei Med. J..

[135] Zhao C.-M., Guo R.-X., Hu F., Chen P.-X., Cui Y., Feng J.-Q., Meng J.-L., Mo L.-Q., Liao X.-X. (2012). Spinal MCP-1 Contributes to the Development of Morphine Antinociceptive Tolerance in Rats. Am. J. Med. Sci..

[136] Lin C.-P., Kang K.-H., Tu H.-J., Wu M.-Y., Lin T.-H., Liou H.-C., Sun W.-Z., Fu W.-M. (2017). CXCL12/CXCR4 Signaling Contributes to the Pathogenesis of Opioid Tolerance: A Translational Study. Anesthesia Analg..

[137] Iellem A., Mariani M., Lang R., Recalde H., Panina-Bordignon P., Sinigaglia F., D’Ambrosio D. (2001). Unique Chemotactic Response Profile and Specific Expression of Chemokine Receptors Ccr4 and Ccr8 by Cd4+Cd25+ Regulatory T Cells. J. Exp. Med..

[138] Juremalm M., Olsson N., Nilsson G. (2002). Selective CCL5/RANTES-induced mast cell migration through interactions with chemokine receptors CCR1 and CCR4. Biochem. Biophys. Res. Commun..

[139] Campbell J.J., Haraldsen G., Pan J., Rottman J., Qin S., Ponath P., Andrew D.P., Warnke R., Ruffing N., Kassam N. (1999). The chemokine receptor CCR4 in vascular recognition by cutaneous but not intestinal memory T cells. Nature.

[140] Kakinuma T., Nakamura K., Wakugawa M., Mitsui H., Tada Y., Saeki H., Torii H., Asahina A., Onai N., Matsushima K. (2001). Thymus and activation-regulated chemokine in atopic dermatitis: Serum thymus and activation-regulated chemokine level is closely related with disease activity. J. Allergy Clin. Immunol..

[141] Vestergaard C., Bang K., Gesser B., Yoneyama H., Matsushima K., Larsen C.G. (2000). A Th2 Chemokine, TARC, Produced by Keratinocytes May Recruit CLA+CCR4+ Lymphocytes into Lesional Atopic Dermatitis Skin. J. Investig. Dermatol..

[142] Cherry J.D., Olschowka J.A., O’Banion M.K. (2014). Neuroinflammation and M2 microglia: The good, the bad, and the inflamed. J. Neuroinflamm..

[143] Perros F., Hoogsteden H.C., Coyle A.J., Lambrecht B.N., Hammad H. (2009). Blockade of CCR4 in a humanized model of asthma reveals a critical role for DC-derived CCL17 and CCL22 in attracting Th2 cells and inducing airway inflammation. Allergy.

[144] Conroy D.M., Jopling L.A., Lloyd C.M., Hodge M.R., Andrew D.P., Williams T.J., Pease J.E., Sabroe I. (2003). CCR4 blockade does not inhibit allergic airways inflammation. J. Leukoc. Biol..

[145] Chvatchko Y., Hoogewerf A.J., Meyer A., Alouani S., Juillard P., Buser R., Conquet F., Proudfoot A.E., Wells T., Power C.A. (2000). A Key Role for Cc Chemokine Receptor 4 in Lipopolysaccharide-Induced Endotoxic Shock. J. Exp. Med..

[146] Lloyd C.M. (2003). Chemokines in allergic airway disease. Curr. Opin. Pharmacol..

[147] Baatar D., Olkhanud P., Sumitomo K., Taub D., Gress R., Biragyn A. (2007). Human Peripheral Blood T Regulatory Cells (Tregs), Functionally Primed CCR4^+^Tregs and Unprimed CCR4^−^Tregs, Regulate Effector T Cells Using FasL. J. Immunol..

[148] Forde E.A., Dogan R.-N.E., Karpus W.J. (2011). CCR4 contributes to the pathogenesis of experimental autoimmune encephalomyelitis by regulating inflammatory macrophage function. J. Neuroimmunol..

[149] Abdul-Majid K.-B., Wefer J., Stadelmann C., Stefferl A., Lassmann H., Olsson T., Harris R.A. (2003). Comparing the pathogenesis of experimental autoimmune encephalomyelitis in CD4^−/−^ and CD8^−/−^ DBA/1 mice defines qualitative roles of different T cell subsets. J. Neuroimmunol..

[150] Galimberti D., Fenoglio C., Comi C., Scalabrini D., De Riz M., Leone M., Venturelli E., Cortini F., Piola M., Monaco F. (2008). MDC/CCL22 intrathecal levels in patients with multiple sclerosis. Mult. Scler. J..

[151] Franciotta D., Zardini E., Ravaglia S., Piccolo G., Andreoni L., Bergamaschi R., Romani A., Tavazzi E., Naldi P., Ceroni M. (2006). Cytokines and chemokines in cerebrospinal fluid and serum of adult patients with acute disseminated encephalomyelitis. J. Neurol. Sci..

[152] Li H., Wang C., Li X., Kong Y., Sun W. (2021). CCL17-CCR4 axis contributes to the onset of vitiligo in mice. Immun. Inflamm. Dis..

[153] Shan J., Shen C., Fang J., Li S., Fan Y. (2019). Potential roles of the CCL17-CCR4 axis in immunopathogenesis of oral lichen planus. J. Oral Pathol. Med..

[154] Baay-Guzman G.J., Bebenek I.G., Zeidler M., Hernandez-Pando R., I Vega M., A Garcia-Zepeda E., Antonio-Andres G., Bonavida B., Riedl M., Kleerup E. (2012). HIF-1 expression is associated with CCL2 chemokine expression in airway inflammatory cells: Implications in allergic airway inflammation. Respir. Res..

[155] Hong L., Wang Q., Chen M., Shi J., Guo Y., Liu S., Pan R., Yuan X., Jiang S. (2021). Mas receptor activation attenuates allergic airway inflammation via inhibiting JNK/CCL2-induced macrophage recruitment. Biomed. Pharmacother..

[156] Ha T.-Y. (2009). The Role of Regulatory T Cells in Cancer. Immune Netw..

[157] Takeuchi Y., Nishikawa H. (2016). Roles of regulatory T cells in cancer immunity. Int. Immunol..

[158] Yang P., Li Q.-J., Feng Y., Zhang Y., Markowitz G.J., Ning S., Deng Y., Zhao J., Jiang S., Yuan Y. (2012). TGF-β-miR-34a-CCL22 Signaling-Induced Treg Cell Recruitment Promotes Venous Metastases of HBV-Positive Hepatocellular Carcinoma. Cancer Cell.

[159] Cheng X., Wu H., Jin Z.-J., Ma D., Yuen S., Jing X.-Q., Shi M.-M., Shen B.-Y., Peng C.-H., Zhao R. (2017). Up-regulation of chemokine receptor CCR4 is associated with Human Hepatocellular Carcinoma malignant behavior. Sci. Rep..

[160] Gao Y., You M., Fu J., Tian M., Zhong X., Du C., Zhu Z., Liu J., Markowitz G.J., Wang F.-S. (2021). Intratumoral stem-like CCR4+ regulatory T cells orchestrate the immunosuppressive microenvironment in HCC associated with hepatitis B. J. Hepatol..

[161] Curiel T.J., Coukos G., Zou L., Alvarez X., Cheng P., Mottram P., Evdemon-Hogan M., Conejo-Garcia J.R., Zhang L., Burow M. (2004). Specific recruitment of regulatory T cells in ovarian carcinoma fosters immune privilege and predicts reduced survival. Nat. Med..

[162] Beyer M., Kochanek M., Darabi K., Popov A., Jensen M., Endl E., Knolle P.A., Thomas R.K., von Bergwelt-Baildon M., Debey S. (2005). Reduced frequencies and suppressive function of CD4+CD25hi regulatory T cells in patients with chronic lymphocytic leukemia after therapy with fludarabine. Blood.

[163] Liu W., Wei X., Li L., Wu X., Yan J., Yang H., Song F. (2017). CCR4 mediated chemotaxis of regulatory T cells suppress the activation of T cells and NK cells via TGF-β pathway in human non-small cell lung cancer. Biochem. Biophys. Res. Commun..

[164] Imai K., Matsuyama S., Miyake S., Suga K., Nakachi K. (2000). Natural cytotoxic activity of peripheral-blood lymphocytes and cancer incidence: An 11-year follow-up study of a general population. Lancet.

[165] Karasaki T., Qiang G., Anraku M., Sun Y., Shinozaki-Ushiku A., Sato E., Kashiwabara K., Nagayama K., Nitadori J.-I., Sato M. (2018). High CCR4 expression in the tumor microenvironment is a poor prognostic indicator in lung adenocarcinoma. J. Thorac. Dis..

[166] Liu Q., Rexiati M., Yang Y., Wang W.-G., Azhati B., Saimaiti W., Wang Y.-J. (2014). Expression of chemokine receptor 4 was associated with poor survival in renal cell carcinoma. Med. Oncol..

[167] Xu M., Wang Y., Xia R., Wei Y., Wei X. (2021). Role of the CCL2-CCR2 signalling axis in cancer: Mechanisms and therapeutic targeting. Cell Prolif..

[168] Ling Z., Li W., Hu J., Li Y., Deng M., Zhang S., Ren X., Wu T., Xia J., Bin Cheng B. (2022). Targeting CCL2-CCR4 axis suppress cell migration of head and neck squamous cell carcinoma. Cell Death Dis..

[169] Thomas J.K., Mir H., Kapur N., Bae S., Singh S. (2019). CC chemokines are differentially expressed in Breast Cancer and are associated with disparity in overall survival. Sci. Rep..

[170] Zhu F., Li X., Chen S., Zeng Q., Zhao Y., Luo F. (2016). Tumor-associated macrophage or chemokine ligand CCL17 positively regulates the tumorigenesis of hepatocellular carcinoma. Med. Oncol..

[171] Al-Haidari A.A., Syk I., Jirström K., Thorlacius H. (2013). CCR4 mediates CCL17 (TARC)-induced migration of human colon cancer cells via RhoA/Rho-kinase signaling. Int. J. Color. Dis..

[172] Silva J.R., Iftinca M., Gomes F.I.F., Segal J.P., Smith O.M.A., Bannerman C.A., Mendes A.S., Defaye M., Robinson M.E.C., Gilron I. (2022). Skin-resident dendritic cells mediate postoperative pain via CCR4 on sensory neurons. Proc. Natl. Acad. Sci. USA.

[173] Williams T.C., Jackson D.J., Maltby S., Walton R.P., Ching Y.-M., Glanville N., Singanayagam A., Brewins J.J., Clarke D., Hirsman A.G. (2021). Rhinovirus-induced CCL17 and CCL22 in Asthma Exacerbations and Differential Regulation by STAT6. Am. J. Respir. Cell Mol. Biol..

[174] Zhang A., Xu Y., Xu H., Ren J., Meng T., Ni Y., Zhu Q., Zhang W.-B., Pan Y.-B., Jin J. (2021). Lactate-induced M2 polarization of tumor-associated macrophages promotes the invasion of pituitary adenoma by secreting CCL17. Theranostics.

[175] Sharma I., Singh A., Sharma K.C., Saxena S. (2017). Gene Expression Profiling of Chemokines and Their Receptors in Low and High Grade Astrocytoma. Asian Pac. J. Cancer Prev..

[176] Mizukami Y., Kono K., Kawaguchi Y., Akaike H., Kamimura K., Sugai H., Fujii H. (2008). CCL17 and CCL22 chemokines within tumor microenvironment are related to accumulation of Foxp3+ regulatory T cells in gastric cancer. Int. J. Cancer.

[177] Panina-Bordignon P., Papi A., Mariani M., Di Lucia P., Casoni G.L., Bellettato C., Buonsanti C., Miotto D., Mapp C., Villa A. (2001). The C-C chemokine receptors CCR4 and CCR8 identify airway T cells of allergen-challenged atopic asthmatics. J. Clin. Investig..

[178] Sebastiani S., Albanesi C., Nasorri F., Girolomoni G., Cavani A. (2002). Nickel-Specific CD4+ and CD8+ T Cells Display Distinct Migratory Responses to Chemokines Produced During Allergic Contact Dermatitis. J. Investig. Dermatol..

[179] Katoh S., Fukushima K., Matsumoto N., Matsumoto K., Abe K., Onai N., Matsushima K., Matsukura S. (2003). Accumulation of CCR4-expressing CD4+ T cells and high concentration of its ligands (TARC and MDC) in bronchoalveolar lavage fluid of patients with eosinophilic pneumonia. Allergy.

[180] Furudate S., Fujimura T., Kambayashi Y., Kakizaki A., Hidaka T., Aiba S. (2017). Immunomodulatory Effect of Imiquimod Through CCL22 Produced by Tumor-associated Macrophages in B16F10 Melanomas. Anticancer Res..

[181] Tanita K., Fujimura T., Sato Y., Lyu C., Kambayashi Y., Ogata D., Fukushima S., Miyashita A., Nakajima H., Nakamura M. (2019). Bexarotene Reduces Production of CCL22 From Tumor-Associated Macrophages in Cutaneous T-Cell Lymphoma. Front. Oncol..

[182] Gao Y., Fan X., Li N., Du C., Yang B., Qin W., Fu J., Markowitz G.J., Wang H., Ma J. (2020). CCL22 signaling contributes to sorafenib resistance in hepatitis B virus-associated hepatocellular carcinoma. Pharmacol. Res..

[183] Tham S.M., Ng K.H., Pook S.H., Esuvaranathan K., Mahendran R. (2011). Tumor and Microenvironment Modification during Progression of Murine Orthotopic Bladder Cancer. Clin. Dev. Immunol..

[184] Peng Z.-Y., Chen R., Fang Z.-Z., Chen B., Wang Z.-H., Wang X.-Y. (2017). Increased local expressions of CX3CL1 and CCL2 are related to clinical severity in lumbar disk herniation patients with sciatic pain. J. Pain Res..

[185] Mordillo-Mateos L., Sánchez-Ramos A., Coperchini F., Bustos-Guadamillas I., Alonso-Bonilla C., Vargas-Baquero E., Rodriguez-Carrión I., Rotondi M., Oliviero A. (2019). Development of chronic pain in males with traumatic spinal cord injury: Role of circulating levels of the chemokines CCL2 and CXCL10 in subacute stage. Spinal Cord.

[186] Fu Z., Jiang Y., Liu J., Lin Z., Jin Y. (2020). Study on plasma CC chemokine ligand 2 level and its promoter region 2518A/G polymorphism in MS patients. Eur. J. Inflamm..

[187] Flores-Villanueva P., Ruiz-Morales J.A., Song C.-H., Flores L.M., Jo E.-K., Montaño M., Barnes P.F., Selman M., Granados J. (2005). A functional promoter polymorphism in monocyte chemoattractant protein–1 is associated with increased susceptibility to pulmonary tuberculosis. J. Exp. Med..

[188] Tucci M., Barnes E.V., Sobel E.S., Croker B.P., Segal M.S., Reeves W.H., Richards H.B. (2004). Strong association of a functional polymorphism in the monocyte chemoattractant protein 1 promoter gene with lupus nephritis. Arthritis Rheum..

[189] Gonzalez E., Rovin B.H., Sen L., Cooke G., Dhanda R., Mummidi S., Kulkarni H., Bamshad M.J., Telles V., Anderson S.A. (2002). HIV-1 infection and AIDS dementia are influenced by a mutant *MCP-1* allele linked to increased monocyte infiltration of tissues and MCP-1 levels. Proc. Natl. Acad. Sci. USA.

[190] Tanuma N., Sakuma H., Sasaki A., Matsumoto Y. (2006). Chemokine expression by astrocytes plays a role in microglia/macrophage activation and subsequent neurodegeneration in secondary progressive multiple sclerosis. Acta Neuropathol..

[191] Granata F., Frattini A., Loffredo S., Del Prete A., Sozzani S., Marone G., Triggiani M. (2006). Signaling events involved in cytokine and chemokine production induced by secretory phospholipase A2 in human lung macrophages. Eur. J. Immunol..

[192] Spoettl T., Hausmann M., Herlyn M., Gunckel M., Dirmeier A., Falk W., Herfarth H., Schoelmerich J., Rogler G. (2006). Monocyte chemoattractant protein-1 (MCP-1) inhibits the intestinal-like differentiation of monocytes. Clin. Exp. Immunol..

[193] Ip W.K., Wong C.K., Lam C.W.K. (2006). Interleukin (IL)-4 and IL-13 up-regulate monocyte chemoattractant protein-1 expression in human bronchial epithelial cells: Involvement of p38 mitogen-activated protein kinase, extracellular signal-regulated kinase 1/2 and Janus kinase-2 but not c-Jun NH2-terminal kinase 1/2 signalling pathways. Clin. Exp. Immunol..

[194] Rantapaa-Dahlqvist S., Boman K., Tarkowski A., Hallmans G. (2006). Up regulation of monocyte chemoattractant protein-1 expression in anti-citrulline antibody and immunoglobulin M rheumatoid factor positive subjects precedes onset of inflammatory response and development of overt rheumatoid arthritis. Ann. Rheum. Dis..

[195] Wang H., Zhang Q., Kong H., Zeng Y., Hao M., Yu T., Peng J., Xu Z., Chen J., Shi H. (2014). Monocyte chemotactic protein-1 expression as a prognosic biomarker in patients with solid tumor: A meta analysis. Int. J. Clin. Exp. Pathol..

[196] Wang K., Gu Y., Liao Y., Bang S., Donnelly C., Chen O., Tao X., Mirando A.J., Hilton M.J., Ji R.-R. (2020). PD-1 blockade inhibits osteoclast formation and murine bone cancer pain. J. Clin. Investig..

[197] Kamei N., Tobe K., Suzuki R., Ohsugi M., Watanabe T., Kubota N., Ohtsuka-Kowatari N., Kumagai K., Sakamoto K., Kobayashi M. (2006). Overexpression of Monocyte Chemoattractant Protein-1 in Adipose Tissues Causes Macrophage Recruitment and Insulin Resistance. J. Biol. Chem..

[198] Gonzalo J.-A., Lloyd C., Wen D., Albar J.P., Wells T., Proudfoot A., Martinez-A C., Dorf M., Bjerke T., Coyle A.J. (1998). The Coordinated Action of CC Chemokines in the Lung Orchestrates Allergic Inflammation and Airway Hyperresponsiveness. J. Exp. Med..

[199] Mahad D.J., Ransohoff R.M. (2003). The role of MCP-1 (CCL2) and CCR2 in multiple sclerosis and experimental autoimmune encephalomyelitis (EAE). Semin. Immunol..

[200] Sato K., Kuratsu J.-I., Takeshima H., Yoshimura T., Ushio Y. (1995). Expression of monocyte chemoattractant protein-1 in meningioma. J. Neurosurg..

[201] Connolly K.A., Belt B.A., Figueroa N.M., Murthy A., Patel A., Kim M., Lord E.M., Linehan D.C., Gerber S.A. (2016). Increasing the efficacy of radiotherapy by modulating the CCR2/CCR5 chemokine axes. Oncotarget.

[202] Chen X., Wang Y., Nelson D., Tian S., Mulvey E., Patel B., Conti I., Jaen J., Rollins B.J. (2016). CCL2/CCR2 Regulates the Tumor Microenvironment in HER-2/neu-Driven Mammary Carcinomas in Mice. PLoS ONE.

[203] Steinberger K.J., Bailey M.T., Gross A.C., Sumner L.A., Voorhees J.L., Crouser N., Curry J.M., Wang Y., DeVries A.C., Marsh C.B. (2020). Stress-induced Norepinephrine Downregulates CCL2 in Macrophages to Suppress Tumor Growth in a Model of Malignant Melanoma. Cancer Prev. Res..

[204] Chiu H.-Y., Sun K.-H., Chen S.-Y., Wang H.-H., Lee M.-Y., Tsou Y.-C., Jwo S.-C., Sun G.-H., Tang S.-J. (2012). Autocrine CCL2 promotes cell migration and invasion via PKC activation and tyrosine phosphorylation of paxillin in bladder cancer cells. Cytokine.

